# Different expression of lipid metabolism-related genes in Shandong black cattle and Luxi cattle based on transcriptome analysis

**DOI:** 10.1038/s41598-020-79086-4

**Published:** 2020-12-14

**Authors:** Ruili Liu, Xianxun Liu, Xuejin Bai, Chaozhu Xiao, Yajuan Dong

**Affiliations:** 1 Laboratory of Animal Physiology and Biochemistry, Animal Embryo Center, College of Animal Science, Qingdao Agricultural University, 700 Changcheng Road, Chengyang, Qingdao, 200109 Shandong People’s Republic of China; 2 Laboratory of Animal Molecular, Shandong Black Cattle Breeding Engineering Technology Center, College of Animal Science, Qingdao Agricultural University, Qingdao, 200109 Shandong People’s Republic of China

**Keywords:** Agricultural genetics, Animal breeding, Genetics, Molecular biology

## Abstract

To provide new ideas for improving meat quality and generating new breeds of cattle, the important candidate genes affecting fat deposition in two kinds of cattle were identified. Eighteen months Shandong black cattle (n = 3) and Luxi cattle (n = 3) were randomly assigned into two environmental. The longissimus dorsi muscles of Shandong Black Cattle and Luxi Cattle were collected and analyzed by fatty acid determination, high-throughput sequencing transcriptomics, qRT-PCR expression profile and western blot. The ratio of unsaturated fatty acids to saturated fatty acids was 1.37:1 and 1.24:1 in the muscle tissues of Shandong black cattle and Luxi cattle, respectively. The results of RNA-Seq analysis revealed 1320 DEGs between the longissimus dorsi of Shandong black cattle and Luxi cattle. A total of 867 genes were upregulated, and the other 453 genes were downregulated. With GO enrichment analysis, it was found that the identified DEGs were significantly enriched in regulation of the Wnt signaling pathway, negative regulation of the Wnt signaling pathway, cAMP metabolic process, fat cell differentiation and among other functions. We found that regulation of lipolysis in adipocytes was the significant enrichment pathway of upregulated genes and downregulated genes, PPAR signaling pathway and AMPK signaling pathway are highly representative pathways of lipid metabolism in Shandong black cattle. Network analysis showed that *PPARGC1A*, *ADCY4*, *ANKRD6*, *COL1A1*, *FABP4*, *ADIPOQ*, *PLIN1*, *PLIN2*, and *LIPE* genes were correlated with key loci genes in multiple metabolic pathways. Meanwhile we found that *FABP4* and *ADIPOQ* had 7 common regulatory factors in different genes, which were *PLIN1*, *PLIN2*, *PPARGC1A*, *RXRA*, *PCK1*, *LEPR*, *LEP*. These genes were involved in regulation of lipolysis in adipocytes, adipocytokine signaling pathway, PPAR signaling pathway. *FABP4* and *ADIPOQ* were selected as important candidate marker genes for fat deposition based on the results.

## Introduction

High-throughput RNA-Seq (RNA-Seq) technology can simultaneously detect the expression of thousands of genes and thus plays an important role in the identification of functional genes and analysis of regulatory networks. In recent years, high-throughput RNA-Seq (RNA-Seq) technology has provided an opportunity to generate large amounts of sequence data for non-model organisms, making it superior to traditional microarray analysis^1,2^. Zhang Yingying^3^ found that 12 genes, including COL1A2, COL1A1, SPP1, MMP2, MYH3, MYH8 and CFD, may be important candidate genes affecting beef quality traits in both sexes of Qinchuan cattle. Matthew McCabe5^4^ used RNA-seq analysis in 2012 to conclude that the negative regulation of liver production in lactating dairy cows was related to fat metabolism; High-throughput RNA-Seq (RNA-Seq) is an emerging technology at the frontier of biological research and has gradually become the focus of research in this field^5,6^.

Shandong black cattle was the first embryo transfer calf to obtain vitrified frozen somatic cell cloned embryos in China, which was the combination of Japanese black cattle, Luxi cattle and Bohai black cattle. First, using the method of a combination of incompletely locked group generations and overlapping generations, the Japanese black cattle were crossed with Luxi cattle and bohai black cattle respectively^7^. Then according to the breeding goal, the new generation core breeding group was established by filial generation of Shandong black cattle. Then the new generation core breeding group hybridized with Shandong black cattle. Finally the second generation group of Japan black cattle (3/4) × luxi cattle (1/4) and the new generation (3/4) × bohai black cattle (1/4) was formed. Then the ideal black cattle and Shandong black cattle were selected from the second generation group and entered the cross-cross fixation stage. In this stage, Several good lines with distant relatives were cross-bred, and then selected and reserved the excellent lines groups. After 4 generations, a new Shandong black cattle variety matching line was finally bred and used as a bull. In 2015, Shandong black cattle was approved by the National Animal and Poultry Genetic Resources Committee as a new population and successfully established a new variety cultivation Chinese base. Luxi cattle is one of the five local beef cattle breeds in China, with high meat production capacity, fresh and tender meat and the reputation of "five flavors and three layers of meat"^8,9^. In this study, Luxi cattle as the first generation of hybrid mother hybridized with Shandong black cattle to improve new breeding varieties. The purpose of our study was to find the changes in transcriptome expression between hybrid cattle and cows, and then to explore the gene expression differences between hybrid offspring and original mother generation from the transcriptome.

In this study, we analyzed the gene regulatory network of fat deposition in the longissimus dorsi muscle of Shandong black cattle and Luxi cattle, identified candidate genes and important metabolic pathways affecting fat deposition, revealed the molecular mechanism affecting fat deposition in beef cattle. New ideas are provided for character improvement and the breeding of new breeds.

## Method

### Experimental animals

The animals used in the experiment, i.e., Shandong black cattle and Luxi cattle, were obtained from Shandong black cattle Technology Co., Ltd., and Dadi Luxi Cattle, respectively. According to the standard NY5127—2002 pollution-free feeding management of beef cattle, feeding Shandong black cattle and Luxi cattle was mainly using green coarse feed and reasonably matched with concentrate feed. Fed three times a day and supplied sufficient drinking water. At the same time, the cattle body should be brushed frequently and the cattle house should be ventilated and dried. Kept clean inside and outside of the cattle house and disinfected once a month. The farm carried on the unified feeding management to the Shandong black cattle and Luxi cattle.

18-Month-old healthy beef cattle were selected, and their bones, muscles and bodies were gradually mature. There are no scratches, scars and scabs on the body surface, and fat deposits in the internal organs and abdomen. No disease was found during examination, and all physiological and biochemical indexes were normal. Specific phenotypic data were shown in Table 1.

**Table 1:** The phenotype data of Shandong black cattle and Luxi cattle.

Variety	Weight (kg)	Body height (cm)	Body length (cm)	Chest circumference (cm)	Cannon circumference (cm)	Thickness of backfat (cm)	Carcass weight (kg)
Shandong black cattle-1	513	137	161	200	20	1.8	418
Shandong black cattle-2	510	135	158	197	19	1.7	411
Shandong black cattle-3	506	131	153	192	18.5	1.7	400
Luxi cattle-1	490	135	147	178	17	1.2	285
Luxi cattle-2	486	130	143	182	15	1.2	270
Luxi cattle-3	492	138	152	173	18	1.3	276

### Sample collection

After acceptance and body measuremengt, live cattle would be slaughtered according to the GBT19477-2004 cattle slaughtering procedures, and then immediately disinfection and anatomy. The longissimus dorsi muscle (located between ribs 12–13 of the thorax) was selected as a sample and divided into small squares of about 2–3 cm^3^. A part of them putted in the cryopreservation tube and the other part putted in 4% paraformaldehyde. Fixed the samples and then marked time, name and number. Finally putted the cryopreservation tube into liquid nitrogen immediately. In the sampling process, spray 75% alcohol on the operating table and in the surrounding environment every 2 or 3 min, try to keep the sterile state, and finally bring the sample back to the laboratory. The samples should be ventilated, dried for preservation and prepared for using.

### Fatty acid determination

The fatty acid methyl ester of animal fat was prepared according to the method in GB/T 9695.2-2008. First, reflux was performed with an appropriate sodium hydroxide methanol solution, 12–15% boron trifluoride methanol solution was added to the solution for reflux, and saturated sodium chloride solution and isooctane were added. The solution was mixed, the extract was obtained, and the isooctane layer was used for analysis on a gas chromatograph. After collecting and entering the fatty acid data of the two groups with Excel 2007, ANOVA was conducted with SPSS 17.0 software. The difference between the two groups was analyzed by one-way ANOVA. The data were expressed as the mean ± standard deviation, with *P* < 0.05 indicating statistical significance.

### RNA extraction and sequencing

Extraction, library preparation and sequencing of total RNA from the longissimus dorsi muscle of Shandong black cattle (B) and Luxi Cattle (L) were performed. Total RNA was extracted by Trizol reagent (Invitrogen) according to the manufacturer's instructions, and genomic DNA was removed by DNase I (Takara). RNA quality (RNA integrity number, RIN) was determined by an Agilent 2100 Bioanalyzer, and a NanoDrop 2000 spectrophotometer (NanoDrop) was used for quantification. High-quality RNA samples (OD260/280 = 1.8–2.2, OD260/230 ≥ 2.0, RIN ≥ 6.5) were used to construct sequence libraries. Illumina Hiseq xten sequencing platform was used in this study. We have stored the RNA sequence data in the public domain GEO NCBI to obtain the GEO accession numbers: GSM4904154, GSM4904155, GSM4904156, GSM4904157, GSM4904158, GSM4904159.

### Read quality control and mapping

The original sequencing data was processed by Fastp software with parameters “‘-Q 20 -P90”’ through the disjointing sequence and low-quality sequence. In this step, reads containing adapter, ploy-N, and low-quality reads were removed from raw data to obtain clean clean reads. The clean reads were aligned to the Bos taurus genome (ARS-UCD1.2) using HISAT v2-2.1.0 software (https://daehwankimlab.github.io/hisat2/) and obtain the orientation pattern. The genome was constructed on the superfast short read mapper Bowtie 2 for mapping with default parameters. All downstream analyses were based on clean, high-quality data.

### Analysis and annotation of differentially expressed genes

To identify the DEGs between the two samples, we used the number of fragments per thousand exons (FPKM) to calculate the expression level of each transcript. The mapped reads of each sample were assembled by StringTie v2.1.3 in a reference-based approach. Cuffdiff tool in Cufflink v2.2.1 was used to calculate FPKMs of coding genes in each sample. Gene FPKMs were computed by summing the FPKMs of transcripts in each gene group. Cuffdiff (http://cufflinks.cbcb.umd.edu/) was used to analyze the differential expression^10^. The selected criteria for DEGs were as follows: the logarithm of fold change was greater than 2, and the q-value (q < 0.05) with FDR correction was less than 0.05. GO (gene ontology) terms were assigned based on the best-hits BLASTx resulted from NR alignments that were derived from Blast2GO v5.2.5 against GO database.

### Analysis of functions and protein–protein interactions (PPIs) of DEGs

To understand the functions of DEGs, goatools (https://pypi.org/project/goatools/) and KOBAS v3.0 (http://kobas.cbi.pku.edu.cn/) were used to conduct analyses of GO functional enrichment and Kyoto Encyclopedia of Genes and Genes (KEGG) pathway enrichment^11,12^. When the q value of a DEG was less than 0.05, the GO and KEGG pathways were considered to be significantly enriched. The GO enrichment analysis for DEGs (hypergeometric test, q-value < 0.05) was performed using clusterProfiler v3.16.0 in the R package.

The PPI network of DEGs was analyzed using the STRING v11 (http://string-db.org/) database, which contains direct and indirect protein associations. Based on the STRING analysis results and expression change information for each DEG, a network diagram of the selected DEGs (connecting one or more DEGs) was drawn by using Cytoscape v3.7.1 software^13^.

### Real-time quantitative PCR (qRT-PCR)

The reaction system (20 μL) consisted of the following: 1 μL of template cDNA, 10 μL each of the upstream and downstream primers, and 5 ml (5 × 1 mL vials) of RNase-free water. The thermal cycling procedure was as follows: 94 °C for 10 min, 94 °C for 30 s, 60 °C for 30 s, and 72 °C for 40 s, with 40 cycles^14^.The expression of GAPDH was calculated by the 2^−△△CT^ method. Primers used for qRT-PCR as shown in Table 2.

**Table 2:** Primers used for qRT-PCR.

Gene	Primer sequence (5′ → 3′)	Product length (bp)	Annealing temperature (°C)
*GAPDH*	F:CCCTTCATTGACCTTCACTACA R:TCCATTGATGACGAGCTTCC	108	61
*FABP7*	F:CAGGACTCAAAGCACATTCAAG R:CTTTGCCATCCCATTTCTGTAC	794	62
*PCK1*	F:CATGACGAGGATGGGCAC R:GTGGGCGATGAGTGTGAG	257	62
*PILN1*	F:GCCAGCACTTCAGACAAAATC R:GGGAGGTTTCTGGGCATC	771	59
*LIPE*	F:TTCTTTCGCACCAGCCACAA R:CGTAGGAAGTCGGCAATTACCCT	161	58
*ADIPOQ*	F:CATTATGACGGCAGCAC R:CAGATGGAGGAGCACAG	192	52
*FABP4*	F:AGTGGGCGTGGGCTTTG R:CTCTTTATGGTGGTTGAT	252	49
*PPARGC1A*	F:TCCCCAGGCAGTAGATCTTC R:TCCTCGTAGCTGTCATACCTG	861	62
*COL6A2*	F:GAGATCCAGGCATCGAAGG R:CCCATCTGTCCCGTTCTTG	761	62
*COL4A5*	F:GAAAGGACCCAGTGGAGTA R:CATTGTGGAGCATCTGTTGT	426	55
*MYLPF*	F:GACCTGCGGGACACTTT R:GTCGGCACCTTTGAGCT	141	56
*MYL3*	F:CACGCCCAAGTGCGAGAT R:CCCTCCACGAAGTCCTCA	219	59
*ADCY1*	F:AGCACTTCCTAATGTCCAACC R:GAAGGCGTAGACATTCCACC	960	62
*ATGL*	F:CCTGCTGATTGCTATGAGTGT R:TCTTTGGAGTTGAAGTGGGTT	101	52
*IPMK*	F:GGATGTAAAGATAGGGCGGAAA R:AAGCACCAAGAACCCAATCT	109	56
*AMPK*	F:TCACCAGTGGACACGAGGAAAG R:CTCAGGTAGCACGGGTCGGAAT	217	56
*AQP7*	F:GCCATCATCTACTTGTTCTT R:GCTGAGGGTCTATCTTCG	176	52

### Western blotting

Muscle tissue samples were placed in a precooled mortar, fully ground into a powder with liquid nitrogen, kept at a low temperature, and transferred to a centrifuge tube containing 1.5 ml of RIPA lysis buffer. The powder and liquid were fully mixed, placed on ice for 30 min, and then vibrated violently every 10 min for 30 s. Then, 5 μL of 5 × SDS was added and suspended, and the mixture was vibrated for 20 s and centrifuged at 3500 r/min for 1 min. After boiling water treatment for 5 min and centrifugation for 5 min at 12,000 r/min, the supernatant, i.e., total protein of muscle tissue, was collected. For specific steps, see^15^. A luminescence kit was used to expose and develop the film in the darkroom. The film negative was scanned, and ImageJ 1.39u was used to analyze the optical density of the target strip.

### Ethics approval and consent to participate

All experimental design and procedures of this study was performed in strict accordance with the recommendations in the Guide for the Care and Use of Laboratory Animals of the National Institutes of Health. The protocol was approved by the Committee on the Ethics of Animal Experiments of Qingdao Agricultural University IACUC (Institutional Animal Care and Use Committee).

## Results

### The phenotype data of Shandong black cattle and Luxi cattle

According to SPSS 22.0(Table 1), the average body weight, body height, body length, chest circumference, cannon circumference, thickness of backfat and carcass weight of Shandong black cattle were 509.67 kg, 134.33 cm, 157.33 cm, 196.33 cm, 19.17 cm, 1.73 cm and 409.67 kg respectively. The average body weight, body height, body length, chest circumference, cannon circumference, thickness of backfat and carcass weight of Luxi yellow cattle were 489.33 kg, 134.33 cm, 147.33 cm, 177.67 cm, 16.67 cm, 1.23 cm and 277.00 kg respectively. The results of correlation coefficient significance test showed that the correlation coefficients between carcass weight and body weight, body height, body length, chest circumference and cannon circumference of Shandong black cattle were 0.999, 0.998, 0.999 and 0.950 respectively, while the correlation coefficients between carcass weight and body weight, body height, body length, chest circumference and tube circumference of Luxi yellow cattle were 0.564 and 0.524 respectively.

### Determination of fatty acids in beef cattle

To confirm the difference in partial fatty acid content between Shandong black cattle (B) and Luxi cattle (L), the fatty acid content of beef was determined. As shown in Table 3, the saturated fatty acid content of Shandong black cattle was 1.34% lower than that of Luxi cattle, and the monounsaturated fatty acid content of Shandong black cattle was higher; the polyunsaturated fatty acid content of Shandong black cattle was significantly higher than that of Luxi cattle. The stearic acid (saturated fatty acid) and linoleic acid (polyunsaturated fatty acid) contents in Shandong black cattle were significantly lower than those in Luxi cattle. The ratio of unsaturated fatty acids to saturated fatty acids in the muscle tissue of Shandong black cattle and Luxi cattle was 1.37:1 and 1.24:1, respectively, and the percentage of monounsaturated fatty acids relative to the total fatty acids of Shandong black cattle was 54.28%. These results confirmed that the beef of Shandong black cattle had a better fatty acid composition and flavor than that of Luxi cattle.

**Table 3:** Difference in the fatty acid content of beef between group B and group L.

Fatty acid component	Shandong black cattle	Luxi cattle
(SFAs) Saturated fatty acids	42.67 ± 4.13	44.01 ± 3.43
(MUFAs) Monounsaturated fatty acids	54.28 ± 3.77	52.11 ± 3.06
(PUFAs) Polyunsaturated fatty acids	3.97 ± 0.55*	2.36 ± 0.33
Myristic acid	2.62 ± 0.45	2.29 ± 0.66
Palmitic acid	23.44 ± 2.80	24.15 ± 2.11
Stearic acid	9.72 ± 0.915*	15.43 ± 4.64
Margaric acid	0.66 ± 0.11	0.57 ± 0.11
Palmitoleic acid	3.24 ± 0.65	3.98 ± 1.27
Myristoleic acid	0.75 ± 0.22	0.93 ± 0.28
Oleic acid	50.90 ± 3.31	47.69 ± 4.76
Peanut olefinic acid	0.38 ± 0.11	0.49 ± 0.12
Linoleic acid	2.52 ± 0.47*	1.06 ± 0.31
Linolenic acid	0.43 ± 0.11	0.45 ± 0.18
Others	3.13 ± 0.69	3.11 ± 0.88

In the table, * indicates a significant difference (*P* < 0.05).

### Transcriptome quantification

After removing the low-quality reads and the reads with adapter sequences from the RNA-Seq raw reads, 223,609,230 clean reads (B: 122,463,284, L: 101,145,946; Table 4) were obtained. In the B and L samples, 98.08% and 97.53% of the reads were located in the reference genome, and the clean (Q30) base rate was 94.30% and 93.72%, respectively.

**Table 4:** Read quality and mapping results for RNA-Seq.

Sample	Total raw reads	Total clean reads	Mapped reads	Mapping rate (%)	Clean (Q30) Base Rate (%)
L1	126,735,736	122,463,284	120,167,801	98.13	94.00
L2	125,664,524	119,811,368	117,518,654	98.09	94.36
L3	125,900,178	119,637,778	117,281,323	98.03	94.54
B1	99,182,298	95,960,692	94,094,172	98.05	93.75
B2	105,345,396	101,145,946	97,711,021	96.60	93.56
B3	102,435,924	98,666,436	96,633,740	97.94	93.86

### DEG analysis and functional annotation

**Figure 1:**
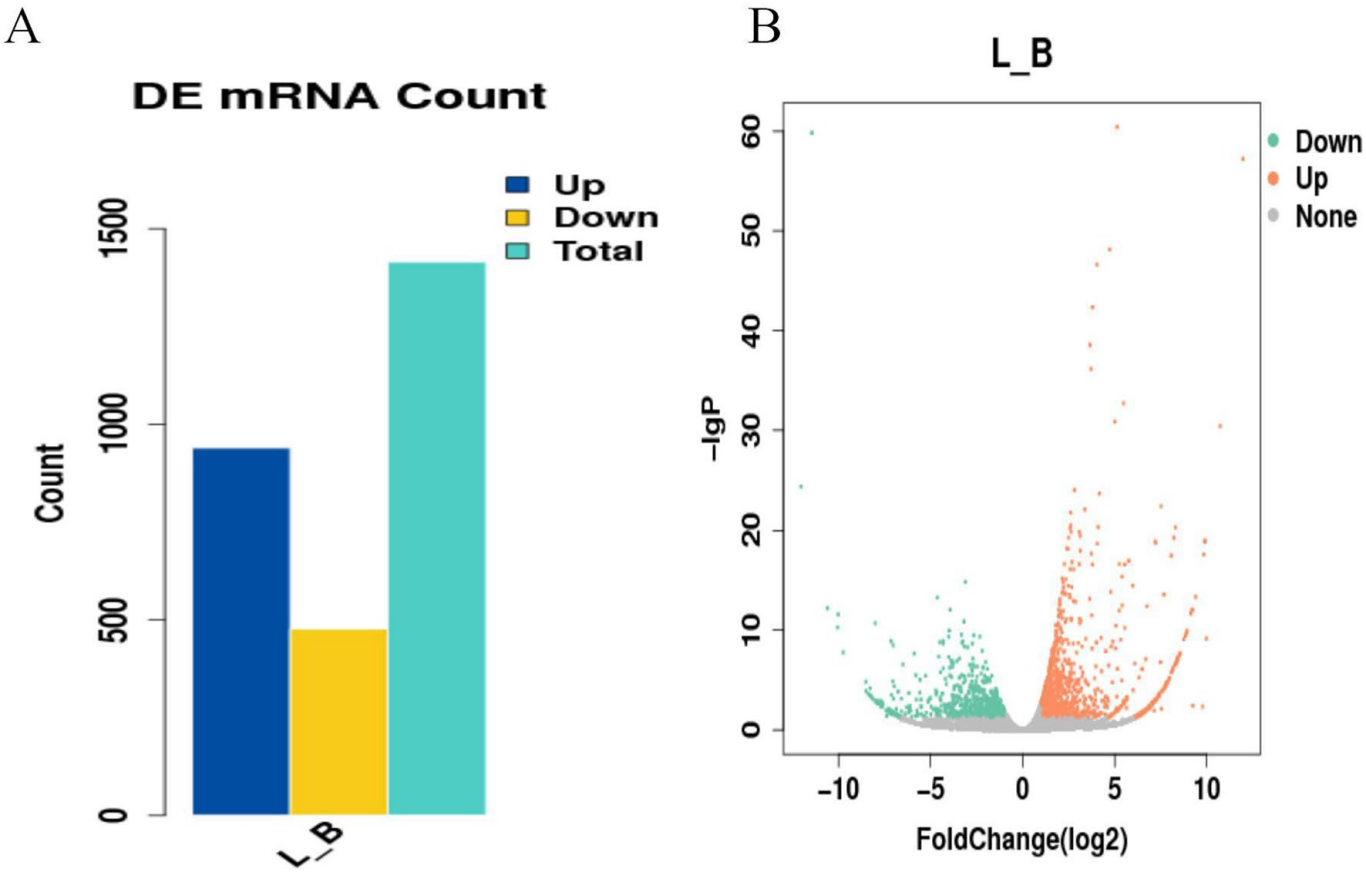
Summary of differentially expressed genes between Shandong black cattle (B) and Luxi cattle (L) **a** A total of 1321 genes were identified as significantly differentially expression, 867 were upregulated, and 453 were downregulated. **b** According to the two variables of fold change and *P*-value, the volcano map was drawn. When the abscissa value is 0, it means that the expression of gene is the same between the two samples, while the ordinate value represents the significant level, and the larger the value is, the higher the significant level is.

For a gene to be considered a DEG, the criteria of an FPKM of l, fold change (FC) ≥  ± 2, and q < 0.05 had to be met. A total of 1320 genes were identified as significantly differentially expressed in the Shandong black cattle group (B). Of the 1320 genes, 867 were upregulated, and 453 were downregulated (Fig. 1; and attachment 1: Fig. S1: Heat map of DEGs between Shandong black cattle and Luxi cattle samples, where yellow indicates upregulation and blue indicates downregulation (PDF, 47 KB); attachment 2: table S1: Differentially expressed genes in Shandong black cattle and Luxi cattle samples (XLS, 111 KB)).

**Figure 2:**
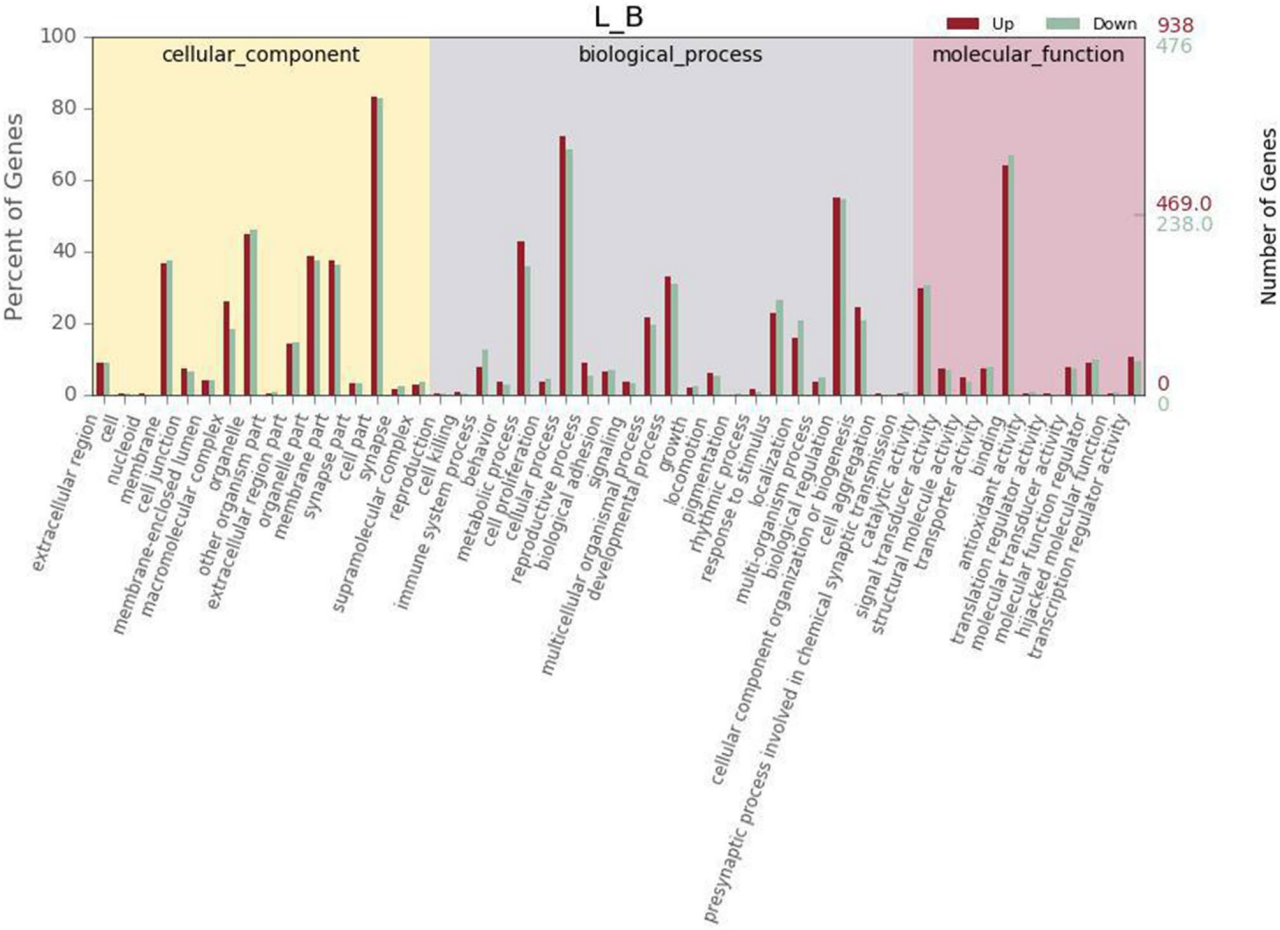
GO annotation of DEGs between the longissimus dorsi muscle of Shandong black cattle and that of Luxi cattle. A total of 1320 DEGs were annotated with 50 different GO terms. The upregulated DEGs were associated with 27 terms, and the downregulated DEGs were associated with 23 GO terms. Red represents upregulated genes, green represents downregulated genes.

In our study, 1320 unigenes were assigned to 50 sub-categories of GO terms belonging to the following three main categories: cellular component (CC), biological process (BP), and molecular function (MF). These main categories included 16, 23, and 11 sub-term categories, respectively (Fig. 2).The DEGs were mainly enriched in the processes of cellular process (BP),biological regulation (BP) of GO sub-categories of biological process (BP) and cell part (CC) of cellular component (CC).The upregulated DEGs were associated with 27 terms, the mainly enriched sub-categories were mainly involved in cell part (CC), cellular process (BP), binding (MF), biological regulation (BP) and metabolism process (BP). The downregulated DEGs were associated with 23 GO terms. The most frequently annotated GO terms were cell part (CC), binding (MF), cellular process (BP), biological regulation (BP) and organelle part (CC).

### Functional and PPI analyses of DEGs

After the enrichment analysis of GO terms, the enrichment ratio of DEGs for different GO terms was analyzed. The DEGs related to fat metabolism were significantly enriched in negative regulation of the Wnt signaling pathway, brown fat cell differentiation, regulation of the Wnt signaling pathway, cAMP metabolic process, fat cell differentiation, biological regulation, regulation of cellular metabolic process, and regulation of primary metallic process, among others (attachment 3: Fig. S2 and attachment 4: Table S2: Results of DEG enrichment analysis between Shandong black cattle and Luxi cattle samples (XLS 159 kb)).

We have analyzed the upregulated genes and downregulated genes respectively for enrichment and other ways, and it is interesting that we find the pathways that upregulated and downregulated genes are significantly enriched is the Regulation of lipolysis in adipocytes. Among the 32 pathways with significant enrichment, focal adhesion, regulation of lipolysis in adipocytes, ECM receiver interaction, AMPK signaling pathway, PPAR signaling pathway and adipocytokine signaling pathway were enriched in typical pathways (www.kegg.jp/kegg/kegg1.html) (attachment 5: Fig. S3, Table 4 and attachment 6: Table S3:KEGG pathway enrichment result of the DEGs.). As a whole significantly enriched lipid metabolism pathway, the upregulated genes is ECM-receptor interaction and Focal adhesion and the downregulated genes is PPAR signaling pathway and AMPK signaling pathway. We regard PPAR signaling pathway and AMPK signaling pathway as a highly representative pathway of lipid metabolism in Shandong black cattle. The differential genes involved in the above pathway are shown in the Table 5.

**Table 5:** List of significantly enriched KEGG pathways related to lipid metabolism.

Pathways	ID	No. of DEGs	*P*-value	Upregulated genes	Downregulated genes
Regulation of lipolysis in adipocytes	Ko04923	13	0.00093	*ADCY4, TAHR, PIK3R3, ADCY1, NPY1R*	*PTGS1, FABP4, PDE3B, ADORA1, ADCY7, LIPE, PLIN1, PTGER3*
Adipocytokine signaling pathway	Ko04920	11	0.039847	*RXRA, SOCS3, NFKBIA, PPARGC1A, ACSBG2*	*PCK1, G6PC, LEPR, SLC2A1, LEP, ADIPOQ*
PPAR signaling	Ko03320	12	0.032492	*PXPA, FADS2, ACSBG2*	*PLIN2, FABP7, PCK1, FABP4, FABP3, PLIN1, GK, ADIPOQ, SCD5*
ECM-receptor interaction	Ko04512	16	0.000853	*COL6A1, COL6A2, LOC530102, COL4A2, ITGA6, LAMA5, COL1A1, HSPG2, DAG1, LAMB2, LAMA3, COL4A6, COL4A5, COL6A3, COL1A2, ITGB8*	
AMPK signaling pathway	Ko04512	18	0.018498	*PFKFB3, CREB3L1, PFKFB2, EEF2K, PIK3R3, PFKM, PPARGC1A*	*ADRA1A, FBP1, PCK1, LIPE, CAB39, G6PC, LEPR, LEP, ADIPOQ, SCD5, PPP2R2C*
Focal adhesion	Ko04510	24	0.017491	*COL6A1, COL6A2, LOC530102, COL4A2, COL4A2, ITGA6, LAMA5, MYLK3, COL1A1, LAMB2, VEGFA, MYLK4, LAMA3, MYLPF, PAK1, COL4A6, COL4A5, PIK3R3, COL6A3, COL1A3, ITGB8, PDGFB, KDR*	*DIAPH1, MYL12A*

**Figure 3:**
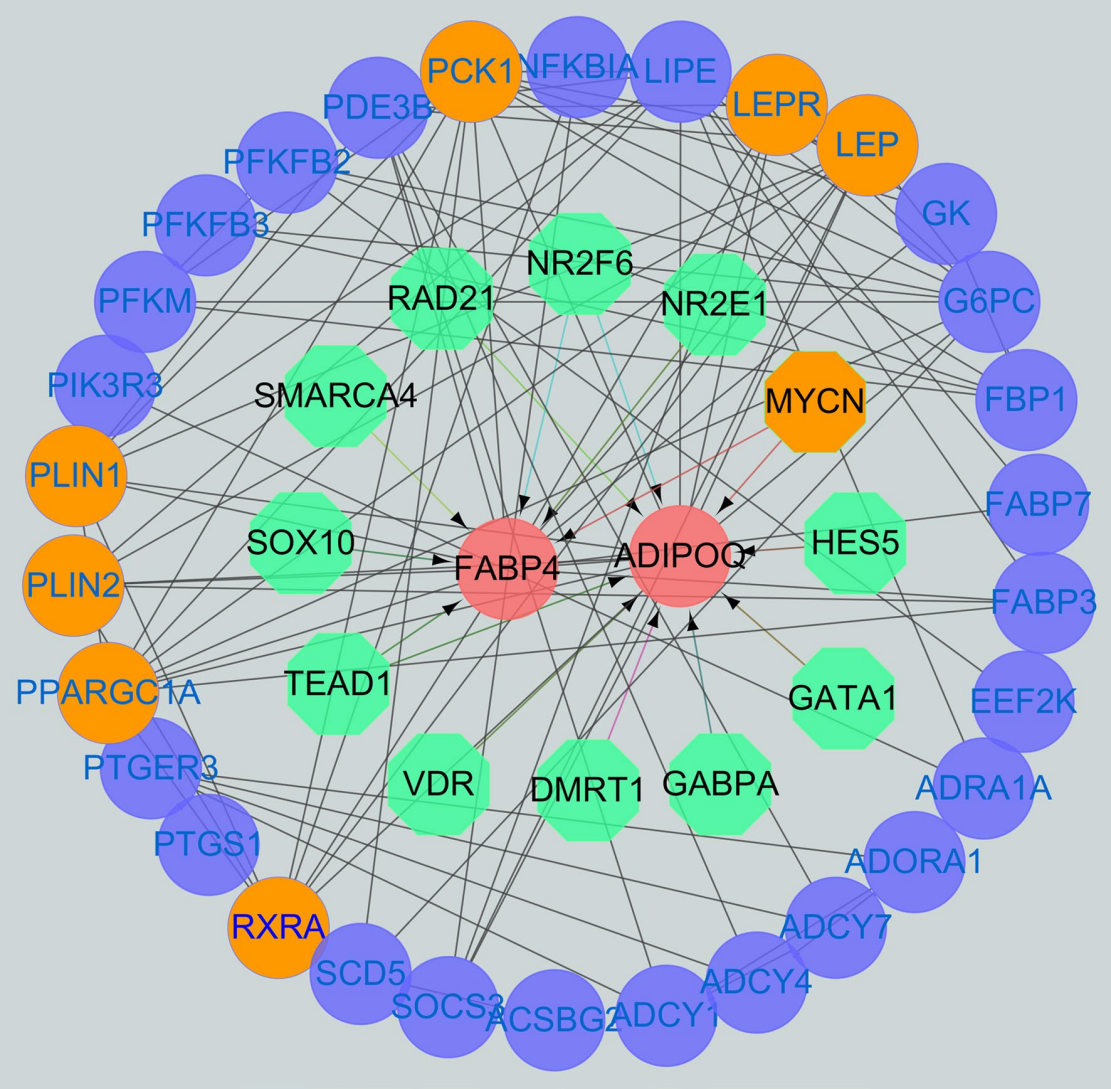
Prediction of differential gene targeting network. Blue represents differential regulatory gene, Green represents predictive regulatory gene, Orange represents the regulator of FABP4 and ADIPOQ genes.

**Figure 4:**
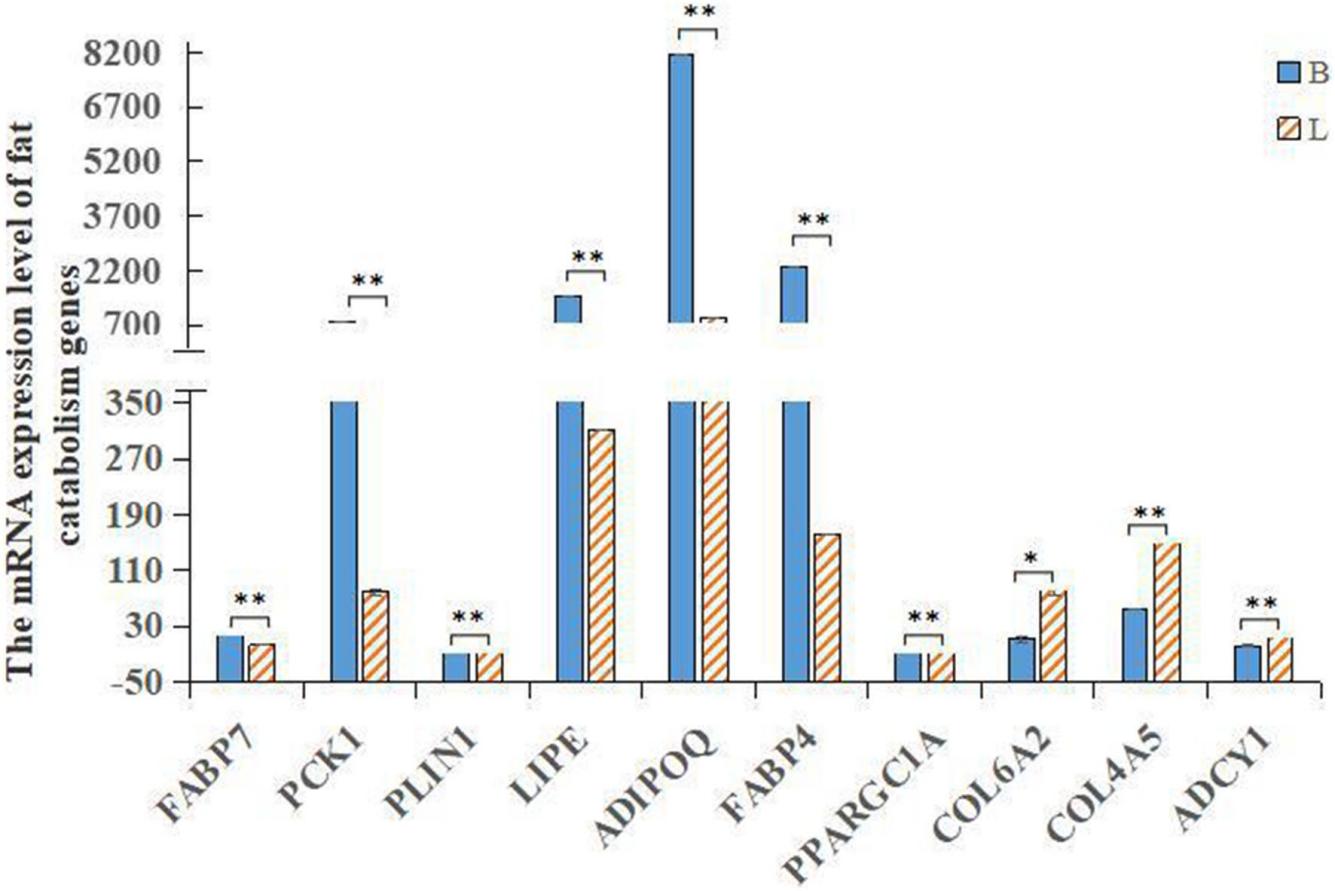
Expression profile of fat metabolism-related genes. The expression of different genes related to fat metabolism in Shandong black cattle and Luxi cattle was determined by qRT-PCR. Data are shown as the mean ± S.D. Columns marked with *(*P* < 0.05) are significantly different from each other, and those with **(*P* < 0.01) show extreme differences.

The potential interaction network between different genes was studied by using STRING analysis. As shown in Fig. 3, most of the DEGs were related to metabolic pathway, focal adhesion, purine metabolism, regulation of lipolysis of adipocytes and the cAMP signaling pathway. Among the upregulated genes, *COL6A1*, *COL6A2*, *LAMB2*, *VEGFA*, *MYLK4*, *LAMA3* and *MYLPF* were located in the core of the network and connected to many other DEGs; among the downregulated genes, the key loci were *FABP4*, *ADIPOQ*, *PLIN1*, *PLIN2* and *LIPE*, which were related to the largest numbers of genes. In addition, not all DEGs were connected to other DEGs because their functions were either not relevant or not yet clarified (attachment 7: Fig. S4:STRING analysis of all DEGs between S2 Shandong black cattle bull and Luxi cattle samples (SVG, 8290 kb)). These DEGs were not included in Fig. 4.

### Expression profile analysis of genes related to fat metabolism

qRT-PCR was used to detect the expression of differentially expressed genes related to fat metabolism. As shown in Fig. 4, most of the DEGs showed significantly different expression (*P* < 0.01), except *COL6A2* (*P* < 0.05). Among the 10 genes related to fat metabolism, *ADIPOQ* exhibited the highest expression in the longissimus dorsi, followed by *FABP4*, *LIPE* and *PCK1*, and the expression of the other genes was relatively low.

### Partial verification of RNA-Seq data

**Figure 5:**
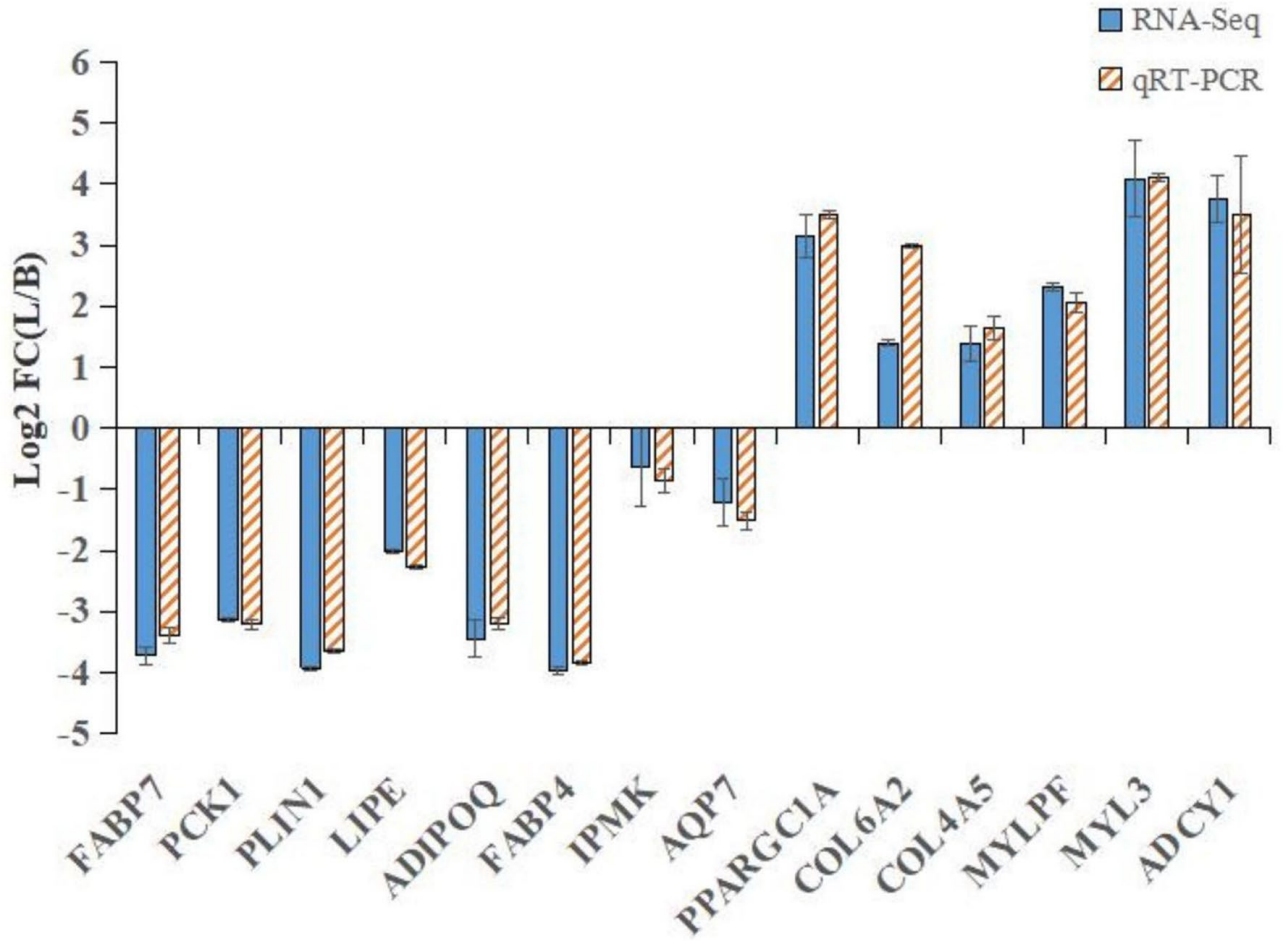
Comparison of gene expression levels detected with RNA-seq and qRT-PCR.

**Figure 6:**
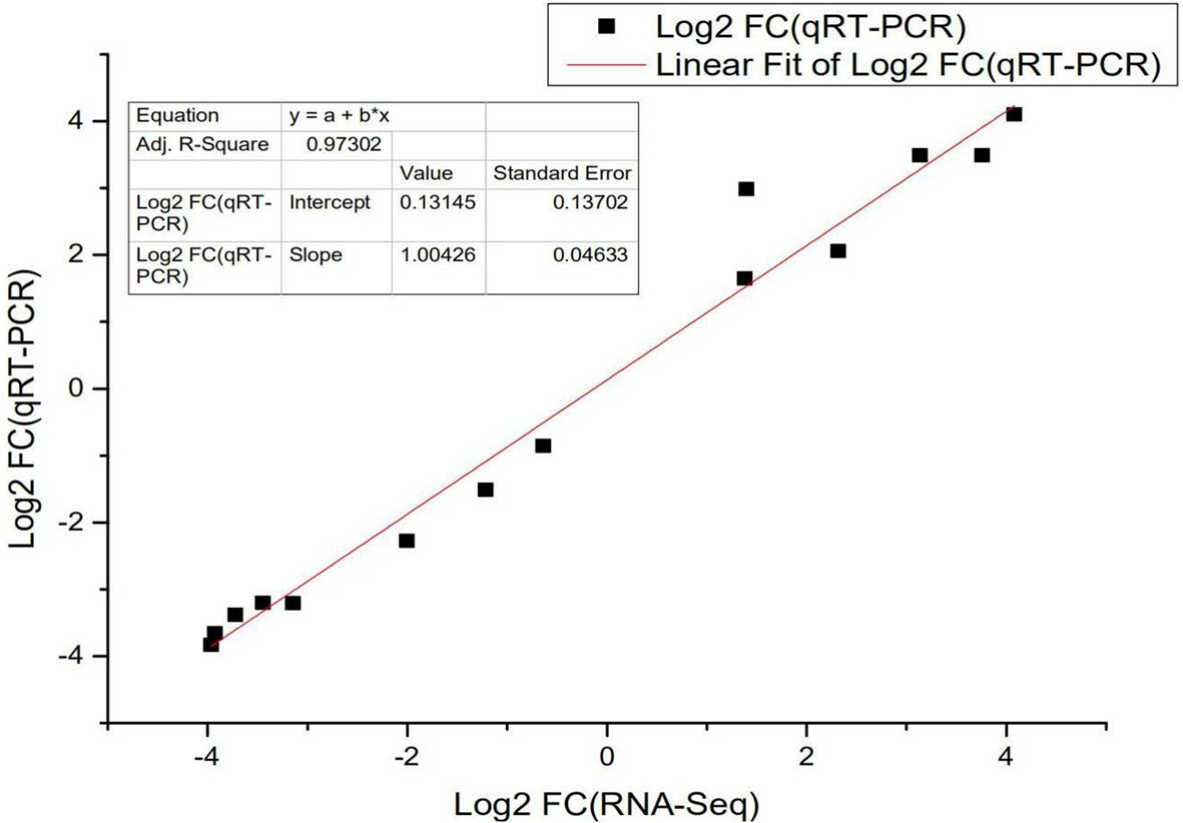
Linear fitting of RNA-seq and qRT-PCR gene expression data**.** The DEGs related to fat metabolism were selected for qRT-PCR analysis. The 14 selected genes showed consistent results between the RNA-seq and qRT-PCR analyses, with a correlation coefficient (R^2^) = 0.97302.

**Figure 7:**
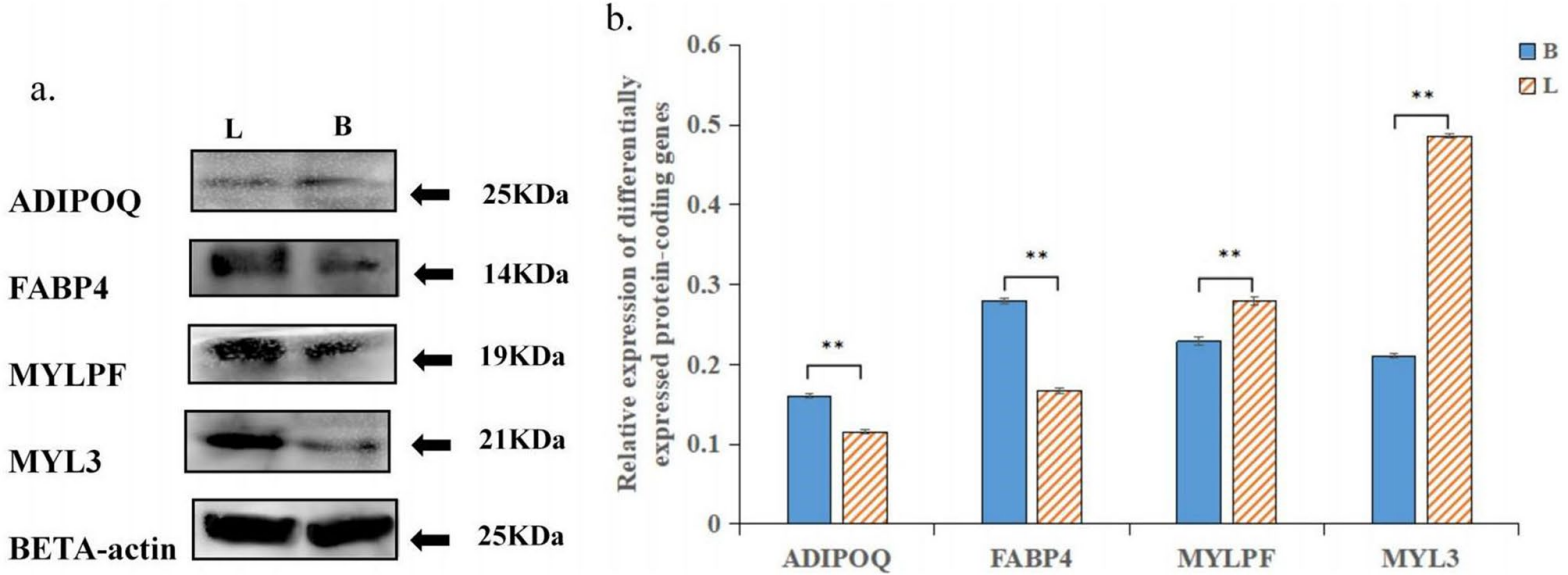
Expression of protein-coding DEGs. **a** The expression of ADIPOQ, FABP4, MYLPF and MYL3 in the samples was determined by Western blot using anti-beta actin polyclonal antibody (Abcam) and anti-rabbit IgG (whole-molecule) antibody (Sigma), unprocessed images are presented in Supplementary Figure S5. **b** The quantitative data of the Western blotting are presented as fold changes compared to the anti-beta actin data after ImageJ quantification; *, ** indicate significant differences among the different treatments (n > 3; *P* < 0.05).

To further verify the RNA-Seq data, the DEGs determined to be related to the fat metabolism response with RNA-Seq were selected for qRT-PCR analysis. As shown in Table 6 and Fig. 5 selected genes showed consistent results between the RNA-Seq and qRT-PCR analyses, and the correlation coefficient between the RNA-Seq and qRT-PCR results (Fig. 6) was very high (R^2^ = 0.97302). Some DEGs were detected by Western blot, as shown in Fig. 7 (attachment 8: Fig. S5: Gel pictures of Western blot). The expression of *ADIPOQ* and *FABP4* decreased significantly (Fig. 7a), and the expression of *MYLPF* and *MYL3 *increased significantly (Fig. 7b). In addition, there was no change in the expression of β-actin (used as a control). The relationship between the Shandong black cattle and Luxi cattle was consistent with the results of RNA-Seq. These results confirmed that the differentially expressed genes identified by RNA-Seq were reliable.

**Table 6:** The immune response-related DEGs and partial validation of RNA sequencing data by qRT-PCR.

Gene ID	Gene name	Functional annotation	FC (log2(L/B))	
			RNA-Seq	qRT-PCR
**Downregulated**				
NM_001078162.2	*FABP7*	Fatty acid binding protein 7	− 3.7202	− 3.3823
NM_174737.2	*PCK1*	Phosphoenolpyruvate carboxykinase 1	− 3.1438	− 3.2106
NM_001083699.1	*PLIN1*	Perilipin 1	− 3.9278	− 3.6566
NM_001080220.1	*LIPE*	Hormone sensitive type	− 2.0047	− 2.2774
NM_174742.2	*ADIPOQ*	Adiponectin, C1Q and collagen domain containing	− 3.4458	− 3.2016
NM_174314.2	*FABP4*	Fatty acid binding protein 4, adipocyte	− 3.9620	− 3.8339
**Upregulated**				
NM_177945.3	*PPARGC1A*	PPARG coactivator 1 alpha	3.1363	3.4889
NM_001075126.1	*COL6A2*	Collagen type VI alpha 2 chain	1.3982	2.9828
XM_002699862.5	*COL4A5*	Collagen type IV alpha 5 chain	1.3815	1.6453
NM_001075647.1	*MYLPF*	Myosin light chain, phosphorylatable, fast skeletal muscle	2.3186	2.0552
NM_001076501.2	*MYL3*	Myosin light chain 3	4.0811	4.1010
NM_174229.2	*ADCY1*	Adenylate cyclase 1	3.7571	3.4890

## Discussion

The main fatty acids that play a role in the flavor of beef are unsaturated fatty acids (UFAs), among which polyunsaturated fatty acids (PUFAs) play the greatest role. The main principle of fatty acid analysis is to determine the relative levels of ω-6 and ω-3 groups in PUFAs. The average ratio of unsaturated fatty acids to saturated fatty acids in beef is 1.37:1, and the percentage of monounsaturated fatty acids (MUFAs) relative to total fatty acids is greater than 55%^16^. The results showed that the ratio of unsaturated fatty acids to saturated fatty acids was 1.37:1 and 1.24:1 in the muscle tissue of Shandong black cattle and Luxi cattle, respectively, and the ratio of monounsaturated fatty acids to total fatty acids was 54.28%. The results showed that the fatty acid composition of Shandong black cattle beef was suitable and that the flavor was good. According to previous research, unsaturated fatty acids can reduce plasma cholesterol, enhance antioxidant enzyme activity, and reduce the incidence of coronary heart disease^17^. A high saturated fatty acid content will lead to arteriosclerosis, narrowing of the arteries and other adverse conditions. The average saturated fatty acid and fatty acid contents in ordinary beef are greater than 50%^18^. A high saturated fatty acid content will lead to an increase in blood lipid concentration^19^. The results showed that the saturated fatty acid content in the two kinds of beef was less than 50%, which was lower than the average level. In mammals, intramuscular fat content was positively correlated with meat color and light and shade^20^. Principal component analysis and sequence clustering analysis also found that beef with higher type 1 fiber contained higher intramuscular fat, saturated fatty acid and monounsaturated fatty acid, which gave us a better understanding of different fatty acid composition in skeletal muscle with different metabolic and stretching characteristics^21^. Therefore, Shandong black cattle has better meat performance and significant improvements compared to Luxi cattle, making it a very good germplasm resource for beef cattle. A large number of studies have reported quantitative trait loci (QTLs) that affect different fat deposition sites in different strains and experimental populations. Theoretically, genes involved in lipid metabolism have potential regulatory effects on intramuscular fat content^22^. However, there are few reports on intramuscular fat related molecular markers (DNA maker)^23^. It is of great significance to search for intramuscular fat related marker genes for modern beef cattle breeding.

In this study, the longissimus dorsi muscle of Shandong black cattle and Luxi cattle was used, and its transcriptome was detected in 18-month-old individuals to understand the overall gene expression changes between Shandong black cattle (offspring of cloned cattle) and Luxi cattle (clone receptor) and to explore the molecular mechanism regulating the metabolism of fat. Recently, Jin et al. used digital expression profile technology to analyze the transcriptome of subcutaneous adipose tissue of individuals with different thicknesses of subcutaneous adipose tissue in two hybrid varieties and found 36 and 152 differentially expressed genes in the two varieties^24^. Nine genes specifically expressed in skeletal muscle related to intramuscular fat deposition have been identified in cattle^25^, but the understanding of fat deposition interaction is not complete. In this study, 1320 genes were identified as significantly differentially expressed in the B group. Among these 1320 genes, 867 were upregulated, and 453 were downregulated. Candidate genes that play an important role in fat metabolism were screened. We founded that the GO functional annotation cluster is divided into three categories: biological process (365 terms), cellular component (45 terms) and molecular function (68 terms), among which biological process accounts for the largest number of genes. We further screened six highly enriched pathways related to fat metabolism, including upregulated genes (*COL6A1, COL6A2, COL4A2, ITGA6, COL1A1, HSPG2, DAG1, LAMB2, LAMA3, COL4A6, COL4A5, COL6A3, COL1A2*) and downregulated genes (*PLIN2, FABP7, PCK1, FABP4, FABP3, PLIN1, GK, ADIPOQ, SCD5*). Previous studies have shown that the upregulated genes *COL5A3*, *COL6A2*, *LAMC2*, *FN1* and *COL3A1* jointly activate *CD44* and then positively regulate the docosanoic acid, palmitic acid and trans-oleic acid contents while negatively regulating the tridecanoic acid, stearic acid and cis-5, 8, 11, 14-eicosapentaenoic acid contents in cells^26^. At the same time, we found that differentially expressed genes(*PLIN2, FABP7, PCK1, FABP4, FABP3, PLIN1, GK, ADIPOQ, SCD5, LEPR, LEP,* ) were enriched in the ECM-receiver interaction pathway, PPAR signaling and focal adhesion pathway, which negatively regulate polyunsaturated fatty acids, stearic acid and linoleic acid (*P* > 0.05). It is worth noting that we found that the significant enrichment pathway of upregulated genes and down regulated genes, PPAR signaling pathway and AMPK signaling pathway are highly representative pathways of lipid metabolism in Shandong black cattle, candidate marker genes affecting lipid metabolism were further screened.

The PPAR signaling pathway plays an important role in regulating lipid metabolism, adipogenesis, insulin sensitivity, inflammatory response, cell growth and differentiation. It has been reported that PPAR family consists of three subtypes PPARα, PPARβ/δ and PPARγ, which play different roles in different tissues and belong to nuclear receptor superfamily ligand activated transcription factors. PPARα was mainly expressed in liver, heart and brown adipose tissue, which was the activator of fatty acid oxidation. PPARβ/δ was widely expressed and played an important role in adipogenic differentiation and adipogenesis^27^. PPARα played an important role in fatty acid oxidation. During the differentiation of preadipocytes, PPARβ/δ preferentially activated PPARγ expression, which was crucial for the subsequent adipogenic differentiation and activation of adipocyte functional genes, such as glucose transporter (GLUT4), fatty acid binding protein (FABP4), adiponectin(*ADIPOQ*), leptin(*LEPR, LEP,*) and other related genes. The known target genes of PPARα (*PLIN1*, *PLIN3*, *LIPE*, and *FABP4*) are related to almost all aspects of lipid metabolism and affect the transcription of various fatty acid transport and metabolism genes, among other roles^28^. FABP4, the known target gene of PPARα, has potential marbling prediction ability among different varieties^29^. In the present study, *ADIPOQ*, *PLIN1*, *PLIN2*, *PLIN3*, *LIPE*, *FABP4*, *FABP3* and *FABP7* were all downregulated, indicating that differences in the above fatty metabolism genes may promote the fatty acid-related metabolism pathway of beef cattle and positively regulate polyunsaturated fatty acids, stearic acid and linoleic acid (*P* > 0.05). The comparative analysis of expression profiles showed that the genes regulating fatty acid biosynthesis, such as *FABP4*, *PLIN*1 and *LIPE*, were highly expressed in Shandong black cattle, while the myogenic genes, namely, *MYL3* and *MYLPF*, were highly expressed in Luxi cattle, which initially revealed the genetic basis of the high intramuscular fat content in Shandong black cattle at the molecular level. These studies show that the degree of intramuscular fat deposition depends on the breed. Combined with transcriptome data analysis, we found that ADIPOQ and FABP4 were expressed in lipolysis regulation, AMPK signaling pathway, adipocytokine signaling pathway and PPAR signaling pathway. Network analysis showed that PPARGC1A, ADCY4, ANKRD6, COL1A1 and FABP4, ADIPOQ, PLIN1, PLIN2 and LIPE genes were related to a large number of genes, which were the key loci of various metabolic pathways. FABP4 and ADIPOQ had 7 common regulatory factors in different genes, which were PLIN1, PLIN2, PPARGC1A, RXRA, PCK1, LEPR, LEP. These genes were involved in regulation of lipolysis in adpocytes, adpocytokine signaling pathway, PPAR signaling pathway. Gene expression appears to provide a simple assay for identification of the source of fatty acid for the deposition of intramuscular fat deposition^30^. FABP4 and ADIPOQ were selected as important candidate marker genes for fat deposition based on the results.

We provided phenotype data of these 6 cattle in method, such as body weight before the kill, body composition and other available measurements. At the same time these data were also fitted to a linear model, and the selected *FABP4* and *ADPIOQ* genes were analyzed for the association of expression amounts. The results are shown in supplement file (attachment 9: Fig. S6 and attachment 10: Fig. S7). In the phenotypic data of the six trial cattle, we found that the body weight, body length, tube circumference, chest circumference, back fat and carcass weight were positively correlated with the expression of *FABP4* and *ADPIOQ* genes. Specially the *FABP4* and *ADPIOQ *had the highest correlation coefficient with body weight and chest circumference, but *FABP4* had stronger correlation with phenotypic data such as body weight and chest circumference.The aim of our study is to select marker candidate genes that affect fat deposition in our bred hybrid cattle. This result is particularly meaningful to us. We will further study this result and dig up the SNP sites that can be labeled. We deeply know that the above results are limited to these 6 trial cattle, which has great limitations on the scientificalness of the experimental results. It is worth noting that q-PCR and western blot in our study only confirmed the expression of RNA-seq identified genes in these 6 cattle, and the sample size had significant limitations on independent validation. Therefore, we will carry out biological verification in the following study to improve the scientific accuracy of this study. On this basis, it is necessary to continue to search for marker sites that can provide gene resources and a theoretical basis for the selection of beef fat traits and the cultivation of new breeds.

Adiponectin (*ADIPOQ*) is a kind of adipocytokine mainly secreted by adipose tissue. Through the action of *ADIPOR1* and *ADIPOR2*, it can activate the signaling pathway of AMPK and PPAR α and inhibit lipid synthesis^31,32^. Recently, researchers also found that some factors (such as *ATGL* and *HSL*) can act downstream of AMPK and mediate the lipid-lowering effect of *ADIPOQ*^33^. The data suggested *ADIPOQ* activated PPARα signaling to increase FGF21 expression and secretion, leading to 1.36 times increase in plasma FGF21 concentration, which suppressed lipolysis in white adipose tissue (WAT). It has been found that ADIPOQ gene plays an important role in lipid metabolism by promoting fatty acid oxidation and inhibiting lipid synthesis^34^. So far, the research on regulating animal lipid metabolism by ADIPOQ gene is mainly focused on human and rodents, but less on livestock and poultry^35^. In the early fattening stage, the expression level of ADIPOQ gene was positively correlated with intramuscular fat content^36^, while it was negatively correlated with intramuscular fat content at the late fattening stage^37^. These results suggest that ADIPOQ gene plays an important role in adipogenesis and metabolism, and may be an important candidate gene affecting intramuscular fat. In this study, the expression profile and protein immunoblotting results showed that the transcription of *ADIPOQ* was downregulated, the expression level in Luxi cattle was significantly lower than that in Shandong black cattle, and there were many DEGs associated with metabolic pathways (regulation of lipolysis in adipocytes, AMPK signaling pathway, adipocytokine signaling pathway and PPAR signaling pathway), which were involved in the process of intramuscular fat regulation and are important candidate genes for the study of livestock meat quality. Based on these findings, further exploration of molecular marker sites in these genes is needed.

*FABP4* was another downregulated DEG found in our study. It not only is involved in the differentiation of fat cells in the PPAR signaling pathway and responsible for the transport of extracellular fatty acids but also plays a role in the lipolysis and fatty acid transport of different tissues^38–40^. Previous studies have shown that the expression of *FABP4* is significantly increased during the process of fat differentiation. In the absence of *FABP4*, the efficiency of fat deposition of adipocytes is significantly reduced^40,41^. Hoashi et al. showed that the i74v mutation in the *FABP4* gene was significantly related to the c16:1 content of fatty acids in 234 Shandong black cattle^42^. Guo studies have shown that FABP4 gene expression can determine the difference of IMF in the two species of cattle and sheep^43^. In 2012, A study of fatty acid composition in the muscle of Korean yellow cattle showed that the c14:0, c16:0 and c20:4 contents were significantly related to the g.3691g > a SNP site of the *FABP4* gene^44^. In this study, *FABP4* was selected as a key candidate gene for improving beef quality. The expression of *FABP4* in the longissimus dorsi muscle of Shandong black cattle was significantly higher than that of Luxi cattle, which will have a significant effect on cattle improvement.

## Conclusions

In conclusion, through RNA-Seq analysis, a series of genes related to fatty acid metabolism were observed in Shandong black cattle and Luxi cattle, and *ADIPOQ* and *FABP4*, which are important candidate marker genes affecting fat deposition, were screened. At the same time, based on the correlation analysis of growth traits of the cattle, high-efficiency breeding for production performance, the establishment of a molecular marker database for local cattle and germplasm resources are expected to result from the findings of this study. The results can also provide a molecular genetic basis for conservation and utilization.

## Supplementary Information

Supplementary Information 1.

Supplementary Information 2.

Supplementary Information 3.

## Data Availability

The datasets generated during and/or analysed during the current study are available from the corresponding author on reasonable request. We have stored the RNA sequence data in the public domain GEO NCBI to obtain the GEO accession numbers: GSM4904154, GSM4904155, GSM4904156, GSM4904157, GSM4904158, GSM4904159.

## Abbreviations

DEGs: Differentially expressed genes
KEGG: Kyoto Encyclopedia of Genes and Genomes
GO: The Gene Ontology
PPI network: Protein–protein interaction network
