# Supplementary material for: Different expression of lipid metabolism-related genes in Shandong black cattle and Luxi cattle based on transcriptome analysis
Source: Sci Rep. 2020 Dec 14;10:21915. doi: 10.1038/s41598-020-79086-4 (PMC7736358; doi:10.1038/s41598-020-79086-4)
Supplement: Supplementary file 1 — Supplementary Information 1. [file 41598_2020_79086_MOESM1_ESM.doc]

**Figure legends:**

**Fig. S1 Heat map of the DEGs between samples**. A total of 1,321 genes were identified as significantly differentially expression, 867 were upregulated, and 453 were downregulated. According to the two variables of fold change and *P*-value, the heat map was visualization using R-Studio(1.3.1093.exe, <https://rstudio.com/products/rstudio/>). Yellow represents up regulation, blue represents down regulation.

**Fig. S2** **GO enrichment analysis of DEGs between samples**. A total of 1320 DEGs were analyzed by go cluster analysis. The horizontal axis represents the name of the sample group, and the vertical axis represents the GO biological terms. Each point indicates the enrichment degree of the GO entry, and the closer the color to red, the higher the enrichment degree. The size of each dot indicates the number of genes enriched in the Go entry. The larger the dot, the more genes are enriched in the GO entry.

**Fig. S3** **The top 32 pathways enriched in the KEGG database for DEGs between samples**. 1320 DEGs were analyzed by go cluster analysis. The horizontal axis represents the name of the sample group, and the vertical axis represents the KEGG biological pathway(www.kegg.jp/kegg/kegg1.html). Each point indicates the enrichment degree of the KEGG entry, and the closer the color to purple, the higher the enrichment degree. The size of each dot indicates the number of genes enriched in the KEGG entry. The larger the dot, the more genes are enriched in the KEGG entry.

**Fig. S4 STRING Analysis of All DEGs between Samples.** visualization of differential gene coding protein interaction. The circle represents a protein and the line represents the interaction. The closer the color is to purple, the more interacting proteins there are.

**Fig. S5** **Gel pictures of Western blot**. The expression of ADIPOQ, FABP4, MYLPF and MYL3 in the samples was determined by Western blot using anti-beta actin polyclonal antibody (Abcam) and anti-rabbit IgG (whole-molecule) antibody (Sigma). In the experiment, according to the size of maker, we cut PVDF membrane and incubate different protein antibodies respectively. In the results, the bands are presented independently.

**Fig. S6 Linear model of phenotypic data and expression of ADIPOQ gene.** We provided phenotype data of these 6 cattle in method, such as body weight before the kill, body composition and other available measurements. At the same time these data were also fitted to a linear model, and the selected ADPIOQ gene was analyzed for the association of expression amounts.

**Fig. S7 Linear model of phenotypic data and expression of FABP4 gene.** We provided phenotype data of these 6 cattle in method, such as body weight before the kill, body composition and other available measurements. At the same time these data were also fitted to a linear model, and the selected FABP4 gene was analyzed for the association of expression amounts.

**Table legends:**

**Table S1 Different expression genes in Shandong black cattle and Luxi cattle**

Gene ID：Gene number of Ensembl database

GeneName：Gene name

Gene：The gene number of this research database

*_count：Reads count of sample *

*_normalize：The normalized results of count values in each group

FoldChange：The normalized multiple relationship between the two groups

Log2FoldChange：The log2 value of the normalized value multiple of the two groups

pval：Calculated p value

padj：P value after correction

Up/Down：Up regulation or down regulation of expression

Significant：significant difference or not

Biotype：Gene type

Position：The coordinates of genes

NR:Seq-id：Optimal results of gene mapped into NR database

NR:Score：Score of gene mapped into NR database

NR:Evalue：The evaluation value of gene mapped into NR database

NR:Description：Functional description of the gene in NR database

NT:Seq-id：The optimal result of gene mapped into NT database

NT:Score：Score of gene mapped into NT database

NT:Evalue：The evaluation value of gene mapped into NT database

NT:Description：Functional description of the gene in NT database

Uniprot:UniProtKB-AC：The optimal results of gene mapped into UniProt database;

Uniprot:Score：Score of gene mapped into UniProt database

Uniprot:Evalue：The evaluation value of gene mapped into Uniprot database

Uniprot:Description：Functional description of the gene in Uniprot database

COG:gene：The gene name was mapped into COG database

COG:Score：Score of gene mapped into COG database

COG:Eval：The evaluation value of gene mapped into COG database

COG:num：The gene ID was mapped into COG database

Pfam:pfam_ID：The gene ID was mapped into Protein family Pfam

Pfam:pfam_Name：The gene name was mapped into Protein family Pfam

Pfam:pfam_Description：Functional description of the gene in Protein family Pfam

GO:biological_process：Gene annotated GO terms of biological processes

GO:cellular_component：Gene annotated GO terms of cellular component

GO:molecular_function：Gene annotated GO terms of molecular function

KEGG:KO：The genes annotated in KEGG

KEGG:Description：Function description in KEGG
